# Annotations

**Published:** 1888-11-24

**Authors:** 


### Annotations.

**Competitive Examinations: A Medical Protest.**

The friends and foes of competitive examinations are having a brisk time of it. The question unhappily, like so many other questions, is not discussed on its merits pure and simple. Judgment is deflected in this way or that by quite other considerations. Many of the enemies of examinations are persons whose teaching has not enabled their pupils to pass so satisfactorily as might have been desired. Many of the friends of competition live and grow rich by preparing young men for the struggle. The subject is almost entirely in the hands of these two classes. We submit that it ought not to be in the hands of either one or the other. They are parties to the suit, and ought on no account to sit on the judicial bench. The real grounds on which the question ought to be settled are two: First, are competitive examinations necessary and beneficial in the public interest? Secondly, are they injurious or otherwise to the bodily and mental health of young men? If they are injurious, is the injury so great as to more than counterbalance the advantage to the public arising from the competitive system? We are well aware that both parties will object to its being made a doctor's question; nevertheless a doctor's question it largely is, without any doubt. It is equally obvious that of authoritative utterances no conclave of doctors has as yet pronounced a single one. Where is the London College of Physicians? What are its ideas on such a pressing question of the hour? Is it wasting its energies on some trumpery detail of etiquette? Where, too, is the General Medical Council? It is but too obvious that both these learned bodies are supremely indifferent to this great, this practical, this urgent public question. It is, as we have said, pre-eminently and almost entirely a problem of physiology and health. It is an infinite pity, from a public point of view, that there is no body of men in whom the country has sufficient faith to refer such a question to. There ought to be such a body. If medicine were really in such a condition of scientific advancement as it ought to be there would be such a body. As individuals, medical men have a recognised and honoured position; as a corporate body nobody recognises their existence. How is this? It is because hardly a medical man in a thousand has as much public spirit as animates the conduct of a village parish clerk. The world, we insist, wants guidance on immediate and urgent questions of physiology and health. Where are its guides?

**Are Women Deteriorating?**

Quite a considerable number of educated women have felt moved to a little opposition by an annotation on "Women and their Victims," which appeared in The Hospital three weeks ago, and which afterwards went the round of the Press. Many of them admitted the general fairness of the statements made, but thought that, on the whole, justice was hardly done to the sex. There was a feeling that the writer was "hard" against women as women. What the writer really felt, and still feels, is that women occupy a position of so much unconscious power in the world that their intellectual and moral character is of supreme importance. To begin with, they are the all-potent spirits of the home, and their way of life is the model and inspiration of all the children and the servants. Not only have they such unlimited charge of children and servants; their husbands also feel the pressure of their influence in a thousand ways, and every day of the week. A little thought will show how vast is the power of women over the child generation, and over their grown-up contemporaries as well. But the real reason for disappointment with the inconsequent conduct of so many modern women is to be found in the very opposite character and life of the best of their sisters. These latter are altogether so noble in heart, in their devotion to high and gentle duty; so free from the vulgar ambition which taints and poisons much of the best work of men; so full of a broad, unecclesiastical, loving, and gentle religion; so self-denying, without pharisaism, and so constant to duty, without the unpleasant self-consciousness which sometimes accompanies duty; indeed, they so fill out the idea of perfection, as the word may be applied to rational and moral creatures, that the comparison of them with women of so inferior a type as the mere creatures of fashion, makes an idealising man deeply angry and perhaps sometimes unjust. The lives of really good women are beyond description, as they are beyond praise. It is this very fact—the knowledge of what some women are, and of what all women might be, that arouses certain men to a strange sternness when they look round them on contemporary social life. Are women degenerating at the present day? On the whole we think not. They never were so well educated as they are now, and they never before showed their capacity for education as they do now. Never were there so many women of proved ability, and never were there so many of high devotion to noble causes. Those who felt that their sex was hardly treated before, will, it is hoped, sympathise with the writer's intentions. The world cannot in the least degree afford to lose that peculiar and beautiful influence which good women exert on all around them. It is the duty and interest of men and women alike, not only to conserve, but to strive to increase that influence by every possible means.

**A "Plaintive Cry."**

There is in the British people a wealth and strength of right feeling which could turn the world upside down if rightly directed. For want of adequate knowledge and clear and authoritative direction this mighty reservoir of pent-up feeling is often as powerless as a mountain lake which has the smallest of outlets and knows no other changes but those of mild evaporation. As a people we do not love injustice, we do not encourage unkindness. Whenever a clear case is made out for us we are willing to make any sacrifice, even the largest, in order that right may be done. Our noble action in the West Indian Slave crisis is often quoted in proof of our national nobility of character, and will continue to be quoted to the end of time. A much less weighty matter of conscience demands our attention at the present time. But though it is less weighty, it is not the less real and genuine. There are thousands of prisons in this land filled with prisoners who have committed no offence, broken no law; who have never known what it was to be charged before judge or jury, or to have an opportunity of saying one single word in their own defence. Yet there they are in different parts of the slums of London and other large towns, small, helpless, unhappy, dirty, and hopeless. These poor little prisoners are the beautiful small birds, which in their natural freedom make the woods and lanes of England merry and musical with their ten thousand various notes. It is impossible to describe the feelings of a lover of the country and of its sights and sounds, when he witnesses the denizens of the trees and hedgerows imprisoned in a wretched and filthy London slum. Messrs. S. W. Partridge and Co. have published a little sixpenny book, called "The Plaintive Cry of Captive Britain." It tells how the pretty little linnet of the orchards and hedgerows has to live, when caught, in a dirty cage five inches long by three inches broad, and three or four inches deep. It is to him, accustomed to the spacious mansion of the open air and the blue skies, a living tomb. The piping bulfinch, whose note and curious ways everybody in the village knows as well as he knows the mewings and pranks of the kitten—he, too, is taken from the tall hedges and freedom of the cow-pasture to a sepulchre of the same size—five inches by three. The tumbler pigeon, that fellow of the most reckless daring, of whom Professor Baldwin is the poorest imitator—he who flies up aloft into the air, and then dashes himself down with folded wings as if he were determined to brain himself a hundred times a-day: this joyous and freedom-loving aëronaut is brought to the "Seven Dials," of all places in the world, and confined in a cage in which he can hardly stand upright without striking his head against the top. And then the dirt, the filth, the stinks (forgive the word), and the ten thousand abominations which disgrace indelibly the wretched inhabitants of those miserable slums! To put all these gentle and beautiful creatures there! It is too brutal; too stupid; an anti-climax which makes one both laugh and cry. We have a practical end in view. A small society should be formed for the protection and deliverance of these helpless prisoners. Lazy, loafing scoundrels of men have no right to satisfy their worthless stomachs and save their idle limbs at the expense of creatures so beautiful, so useful, and so defenceless as these.

